# Dynamics and Determinants of Pneumococcal Antibodies Specific against 13 Vaccine Serotypes in the Pre-Vaccination Era

**DOI:** 10.1371/journal.pone.0147437

**Published:** 2016-01-21

**Authors:** Annemarijn C. Prins-van Ginkel, Guy A. M. Berbers, Lucienne H. Grundeken, Irina Tcherniaeva, Jelle I. Wittenberns, Karin Elberse, Liesbeth Mollema, Hester E. de Melker, Mirjam J. Knol

**Affiliations:** Centre for Infectious Disease Control, National Institute for Public Health and the Environment, Bilthoven, The Netherlands; Melbourne School of Population Health, AUSTRALIA

## Abstract

**Introduction:**

Introduction of pneumococcal conjugate vaccines (PCVs) for infants decreased overall invasive pneumococcal disease (IPD), while non-vaccine serotype IPD increased. To fully understand this serotype replacement, knowledge about serotype dynamics in the pre-vaccine era is needed. In addition to IPD surveillance and carriage studies, the serotype replacement can be investigated by serosurveillance studies. The current study compared the results of two Dutch serosurveillance studies conducted in 1995–1996 (PIENTER1) and 2006–2007 (PIENTER2).

**Methods:**

Participants in these studies donated a blood sample and completed a questionnaire. Pneumococcal antibodies of serotypes included in PCV13 were measured with a fluorescent-bead based multiplex immunoassay. Geometric mean antibody concentrations (GMCs) and determinants of pneumococcal antibody levels were investigated.

**Results:**

GMCs were higher in PIENTER2 for serotypes 1, 6A, 6B, 9V, 18C, 19F and 23F and lower for 3 and 5. Age, day care attendance, household size, vaccination coverage, and urbanisation rate were associated with pneumococcal antibodies in children. Education level, ethnicity, age, low vaccination coverage sample, urbanisation rate, and asthma/COPD were associated with pneumococcal antibodies in elderly. The determinants significantly associated with pneumococcal IgG were slightly different for the elderly in PIENTER1 compared to the elderly in PIENTER2.

**Conclusion:**

Although most of the serotype antibody levels remained stable, some of the serotype-specific antibody levels varied during the pre-vaccine era, indicating that exposure of certain serotypes changes without interference of PCVs.

## Introduction

*Streptococcus pneumoniae* is an important cause of meningitis, pneumonia and bacteraemia in young children and elderly [1–3]. The pneumococcus is a common resident of the nasopharynx of humans and especially in children. Colonisation can precede transmission from human to human, an antibody response against the colonising serotype, and development of pneumococcal disease [4]. Children are the most important reservoir of this pathogen; they can transmit the pneumococcus to other children, adults and elderly [4].

In order to prevent invasive pneumococcal disease (IPD) in children, many countries have added the pneumococcal conjugate vaccine (PCV) to their national immunisation program (NIP) [1–3]. The PCVs currently target a maximum of 13 serotypes, while >90 serotypes are known [4]. Vaccination of infants blocks transmission of vaccine serotypes to other age groups. PCVs are highly effective in preventing IPD caused by the vaccine serotypes, but the number of IPD cases caused by non-vaccine serotypes has been rising [1, 5].

In order to understand the effects of vaccination on the distribution of serotypes causing pneumococcal disease, it is important to investigate the dynamics of the different pneumococcal serotypes in the pre-vaccine era. Knowledge on serotype specific transmission over time provides information about the potential spread of non-vaccine serotypes now colonizing the infants. Also, it is relevant to investigate whether the risk factors for acquiring the pneumococcus change over time independently of vaccination to better interpret possible changes after introduction of vaccination.

Serosurveillance studies conducted at different points in time in the pre-vaccine period could shed light on possible changes in the antibody levels in the absence of vaccination. Such studies provide a base line measurement before the vaccine implementation and could help to evaluate the effects observed after implementation. The presence of antibodies shows that the individual has at least once encountered the specific serotype and the serotype was able to induce an antibody response. While carriage studies provide important insights in the prevalence of serotypes in the nasopharynx they provide often more a snapshot. Also, serosurveillance studies allow for the measurement of a high number of subjects.

In this study we compared the results of two serosurveillance studies conducted in the Netherlands in 1995–1996 and 2006–2007, with the aim of eventually comparing these results with post-vaccine studies for both carriage and serosurveillance. The 7-valent PCV was added to the NIP in April 2006 and no national catch-up campaign was organized. Therefore almost all participants were not vaccinated with PCV except a small group of 0–1 year old children of whom only 9 received the booster vaccination [6]. We investigated changes in pneumococcal antibody levels over time and we assessed determinants for these levels. Our hypothesis was that the pneumococcal antibody levels might change over time when comparing the two serosurveillance studies in the pre-vaccine era and that the determinants of antibody levels were the same for both studies.

## Methods

### Study design

The current study used data of two population-based cross-sectional serosurveillance studies conducted in the Netherlands in 1995–1996 and 2006–2007 (PIENTER1 and PIENTER2). The main aim of the PIENTER1 and PIENTER2 studies was to evaluate the seroepidemiology of diseases targeted by the NIP. PIENTER1 was approved by the Medical Ethical Committee of Netherlands Organisation for Applied Scientific Research (TNO) in Leiden [7]. PIENTER2 was approved by the medical ethics testing committee of the foundation of therapeutic evaluation of medicines (METC-STEG) in Almere (clinical trial number: ISRCTN 20164309) [6, 8, 9]. All participants gave written informed consent. For the children, written informed consent was obtained from the parents or legal guardians. Elderly and children with parents/legal guardians who had the ability to give written informed consent were included in the study.

**Fig 1:**
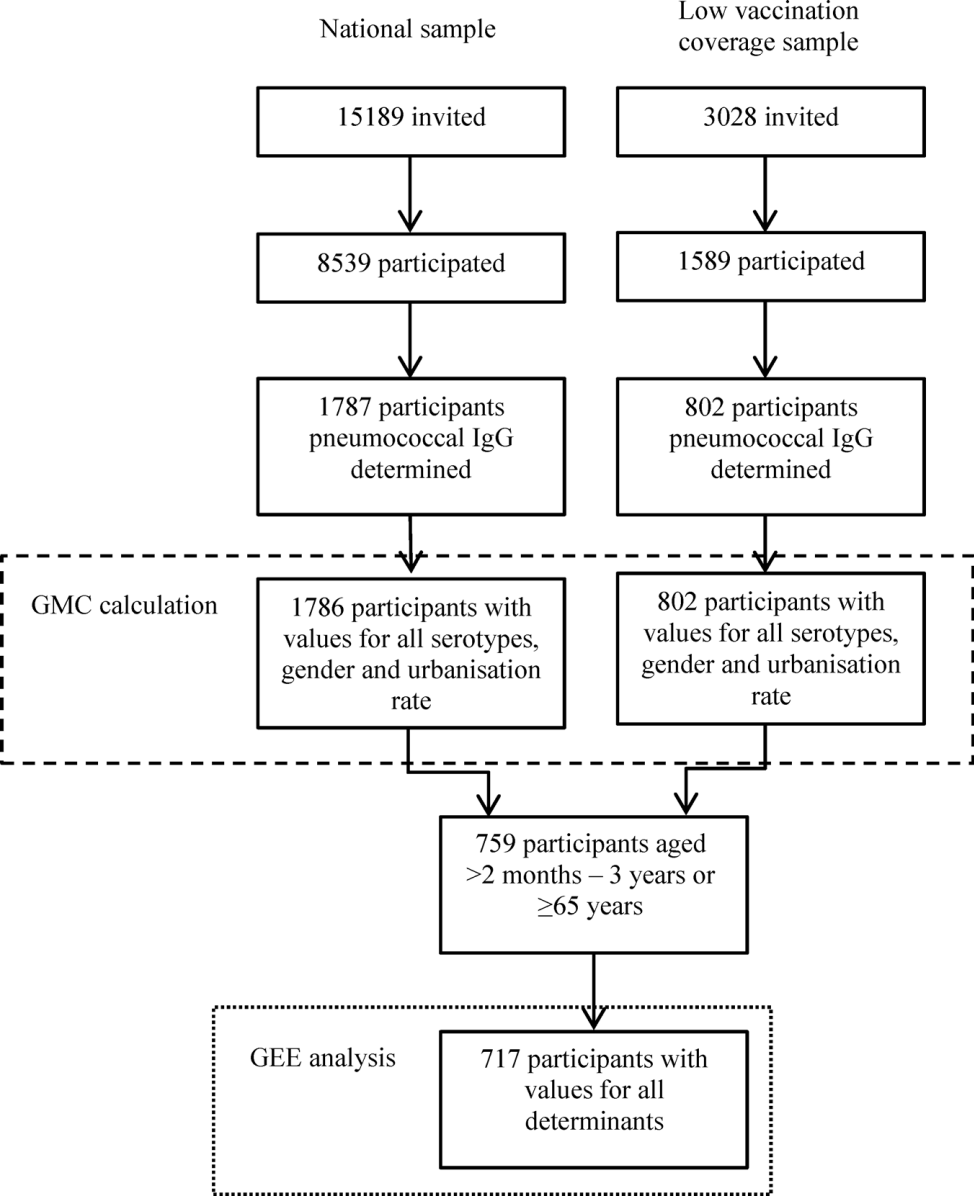
Flowchart of the PIENTER1 study population. The number of persons invited to participate in the PIENTER1 study and the number actually included in the GMC ratio (dashed line) and the determinant calculations (dotted line).

**Fig 2:**
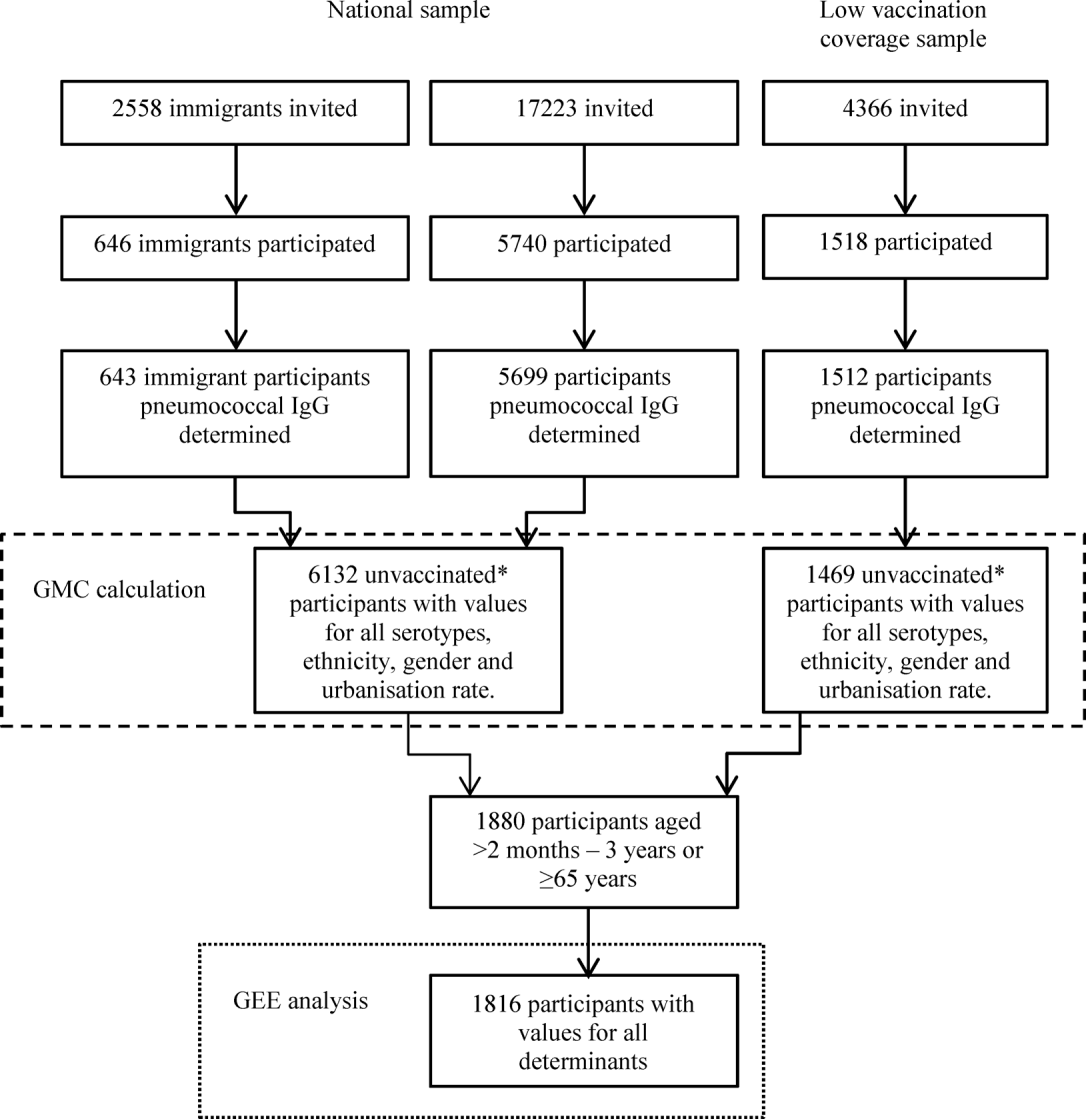
Flowchart of the PIENTER2 study population. The number of persons invited to participate in the PIENTER2 study and the number of participants actually included in the GMC ratio (dashed line) and the determinant calculations (dotted line). * Participants not vaccinated with a pneumococcal conjugate vaccine (PCV), participants vaccinated with other (non-pneumococcal) vaccines were not excluded.

The two studies took a representative sample of the Dutch population. Both had approximately the same study design, which are described in detail elsewhere [7, 8]. In short, subjects, aged 0–79 years, were sampled from the municipal population register using a two-stage cluster sampling technique, which resulted in the national samples (NS). In PIENTER2 oversampling of non-western immigrant populations was performed. In both studies, separate samples of low vaccination coverage (LVC) areas were taken, but different strategies were used. LVC areas were characterised by a vaccination coverage of less than 80% for four DTP-IPV immunizations in children of 2 years of age, this in contrast to the national average coverage of 96% [6, 7, 9]. The PCV was added to the NIP in April 2006, and was therefore not part of the vaccination coverage calculation. All subjects were asked to complete a questionnaire and to donate a blood sample. Of the 18,217 persons who were invited to participate in the PIENTER1 study, 10,128 (56%) donated a blood sample. In PIENTER2 24,147 persons were invited and 7904 (33%) donated a blood sample (Figs 1 and 2) [6–9].

The current study compared the results of the PIENTER2 study with the results of the PIENTER1 study with respect to geometric mean concentration (GMC) and determinants of pneumococcal antibody concentrations. Pneumococcal antibody concentrations were available for 2589 participants of PIENTER1, which was a random sample stratified for age groups of participants for whom a blood sample was available, and for 7854 participants of PIENTER2.

### Determinants

The selection of determinants investigated in the current study was based on previous studies [4, 10–12] and the availability of determinants in the PIENTER1 and PIENTER2 questionnaires; not all determinants were available in both studies. The determinants were subdivided into three groups, namely contact patterns, exposure factors, and sociodemographic characteristics.

Contact patterns were determined by asking whether the subject had contact with another person (excluding household members) and the number of contacts on the day before filling out the questionnaire. Contact was defined as skin-to-skin, a two-way conversation with ≥3 words in the physical presence of another person, or playing with a friend. The contacts were stratified into 4 groups according to the age of the contacted person: 0–4, 5–19, 20–59, and ≥60 years. Within each age stratum the number of contacts was categorized in either 2 categories (contact and no contact) or 3 categories (>5 contacts, 1–4 contacts and no contact).

Four exposure factors were available in the PIENTER2 study, namely day care attendance (0, 0.5–2 and >2 days per week), having a household member of ≤4years (no/yes), the number of household members (1–2 vs. >2 for participants ≥65 years, and 1–4 vs >4 for children aged 2 months- 3 years) and having an occupation or doing volunteer work involving children (no/yes).

Sociodemographic characteristics included age (in years), gender and presence of asthma or COPD. Ethnicity was categorized in western and non-western. Western ethnicity was defined as the participant or one or both parents of the participant were born in a western country. The level of education of the (parents of the) participant was stratified into low (primary school), middle (completed one of the first two levels of secondary education) and high (completed the last two levels of secondary education or higher). Urbanisation rate was categorized based on postal code density, very high (>2500 addresses per km), high (1500–2500 addresses per km), moderately high (1000–1500 addresses per km), low (500–1000 addresses per km) and very low (<500 addresses per km).

Persons were asked to check a box when they had contact with other persons the day before the questionnaire was filled out, when they had asthma or COPD and when they had an occupation involving children. All persons that did not check the box were included in the no category.

### Laboratory methods

In both PIENTER1 and PIENTER2 the pneumococcal IgG antibody concentrations of serotypes included in PCV13 were measured with a fluorescent-bead based multiplex immunoassay as described earlier by Elberse et al. [13]. In short, sera were diluted 1:100 (and sometimes 1:1000) in buffer containing 15 μg/ml cell wall polysaccharide Multi (Statens Serum Institute, Copenhagen, Denmark) and 5% antibody depleted human serum to reduce non-specific reactions. An in-house reference serum calibrated on lot 89-S serum was diluted twofold in 12 steps and included on each plate together with 3 control sera. All capsular polysaccharides were obtained from the American Type Culture Collection (ATCC, Manassas, VA) except for polysaccharide 6A which was kindly provided by Pfizer Inc (New York, NY). For each sample, median fluorescent intensity was converted to IgG concentration (μg/ml) by interpolation from a 5-parameter logistic standard curve. Results were obtained using a Bio-plex 200 system with Bio-plex software (version 6.1, Bio-Rad, UK).

### Data analysis

Participants for whom information about gender, age and urbanisation rate was available, pneumococcal antibody concentrations for all 13 serotypes were determined, and who were not vaccinated with PCV were included in the GMC calculations (1786 of PIENTER1 and 6132 of PIENTER2 of the NS and 802 of PIENTER1 and 1469 of PIENTER2 of the LVC samples, Figs 1 and 2). We calculated age- and serotype-specific GMCs with corresponding 95% confidence intervals (CI). For the NS sampling weights were calculated based on urbanization rate, age and gender and for PIENTER2 also on ethnicity to correct for the oversampling of non-western immigrants. For the LVC samples, sampling weights were calculated based on age and gender. These weights were incorporated in the calculation of the GMC. A GMC ratio with 95% CI was calculated to compare PIENTER2 with PIENTER1; the 95% CI was calculated according to the method described by Altman and Bland [14].

Generalized estimating equations (GEE) analysis was used to estimate the associations between the determinants and the pneumococcal antibody concentrations. The analyses estimating the associations between determinants and pneumococcal antibody concentrations were performed for children and elderly since these are the most important age groups at risk for IPD. The GEE analyses were performed for children aged 0–3 years and elderly of ≥65 years in separate analyses. Participants were included in the GEE analysis when all determinants and pneumococcal antibody concentrations for all 13 serotypes were available, when they were not vaccinated with PCV, and when they were older than 2 months of age (when maternal antibodies should have disappeared). For PIENTER1 372 children and 345 elderly and for PIENTER2 642 children and 1174 elderly were included. (Figs 1 and 2) GEE analysis was used to obtain both a GMC ratio per serotype and an overall GMC ratio across all serotypes for each determinant, both with corresponding 95% CI. The GMC ratios for each determinant per serotype were examined and the highest and lowest estimate formed the range of GMC ratios presented in Tables 1 and 2. The overall GMC ratios across all serotypes are presented, since we did not found the association between determinant and pneumococcal antibody levels to vary majorly between serotypes. The crude model of the GEE analysis yielding overall estimates contained two variables namely the determinant of interest and serotype. An interaction term between the determinant of interest and serotype was included to evaluate whether the effect of the determinant differed between serotypes. The adjusted model contained serotype and all available determinants and interaction terms (each determinant*serotype). When the overall estimate of a specific determinant was calculated the interaction term (specific determinant*serotype) was left out of the model. The results of the GEE analysis yielding an estimate per serotype were used to present the lowest and highest serotype specific GMC ratio for the adjusted model. For the GEE analyses an exchangeable working correlation structure was used, except when the model was not able to calculate all estimates, in that case an unstructured working correlation structure was used.

**Table 1:** Geometric mean antibody concentration (GMC) ratios of children aged 0–3 years PIENTER1 and PIENTER2 based on univariable and multivariable generalized estimating equation analysis.

	PIENTER1					PIENTER2				
Determinants	Crude GMC ratio [95%CI]	P-value interaction- term crude analysis	Adjusted GMC ratio [95% CI]	P-value interaction-term adjusted analysis	Range of GMC ratio’s	Crude GMC ratio [95%CI]	P-value interaction- term crude analysis	Adjusted GMC ratio [95% CI]	P-value interaction-term adjusted analysis	Range of GMC ratio’s
**Contact with 0–4 year olds**	NA	NA	NA	NA	NA		0.005		0.087	
Many contact (>5persons)						**1.60[1.3–2.0]**		1.11[0.9–1.4]		0.91–1.61
Average contact (1–4 persons)						**1.25[1.0–1.5]**		1.10[0.9–1.3]		0.95–1.25
No contact						Ref.		Ref.		Ref.
**Contact with 5–19 year olds**	NA	NA	NA	NA	NA		0.166		0.629	
Many contact (>5 persons)						1.38[1.0–1.9]		0.95[0.7–1.3]		0.75–1.59
Average contact (1–4 persons)						**1.29[1.1–1.5]**		0.98[0.8–1.2]		0.83–1.22
No contact						Ref.		Ref.		Ref.
**Contact with 20–59 year olds**	NA	NA	NA	NA	NA		0.541		0.595	
Many contact (>5 persons)						1.21[0.9–1.6]		0.94[0.8–1.2]		0.74–1.18
Average contact (1–4 persons)						**1.28[1.1–1.5]**		1.12[0.9–1.4]		0.94–1.31
No contact						Ref.		Ref.		Ref.
**Contact with 60 +year olds**	NA	NA	NA	NA	NA		0.131		0.057	
Contact						1.11[0.9–1.4]		0.98[0.8–1.2]		0.83–1.15
No contact						Ref.		Ref.		Ref.
**Household member** ≤ **4 years**	NA	NA	NA	NA	NA		0.721		0.735	
Participant and cohabitant ≤ 4 years						0.88[0.8–1.1]		0.89[0.8–1.0]		0.83–0.98
Only participant ≤ 4years						Ref.		Ref.		Ref.
**Frequency of day care attendance**		<0.001		**0.019**			<0.001		0.212	
>2 days	**1.80[1.2–2.8]**		1.34[0.9–2.0]		0.84–2.15	**1.58[1.2–2.0]**		1.22[0.9–1.6]		0.85–1.71
0.5–2 days	**2.42[2.0–2.9]**		**1.36[1.1–1.7]**		0.90–2.23	**1.62[1.4–1.9]**		1.14[1.0–1.4]		0.88–1.50
No attendance	Ref.		Ref.		Ref.	Ref.		Ref.		Ref.
**Household size**		0.335		0.170			0.046		**0.007**	
1–4 persons	**0.76[0.6–0.9]**		0.84[0.7–1.0]		0.68–1.07	**0.78[0.7–0.9]**		**0.76[0.7–0.9]**		0.60–0.91
>4 persons	Ref.		Ref.		Ref.	Ref.		Ref.		Ref.
**Gender**		0.953		0.863			0.367		0.283	
Male	**0.82[0.7–1.0]**		0.86[0.7–1.0]		0.75–1.00	0.96[0.8–1.1]		1.00[0.9–1.2]		0.87–1.21
Female	Ref.		Ref.		Ref.	Ref.		Ref.		Ref.
**Age**	**1.56[1.4–1.7]**	<0.001	**1.42[1.3–1.6]**	**0.022**	1.23–1.71	**1.57[1.5–1.7]**	<0.001	**1.52[1.4–1.6]**	**<0.001**	1.31–1.99
**Ethnicity**		<0.001		**0.007**			0.292		0.664	
Non-Western	1.12[0.7–1.7]		1.04[0.7–1.6]		0.67–2.44	**1.39[1.1–1.8]**		1.20[0.9–1.6]		1.02–1.67
Western	Ref.		Ref.		Ref.	Ref.		Ref.		Ref.
**Level of education**		<0.001		0.075			0.025		0.054	
Low	**1.39[1.0–1.9]**		1.01[0.5–1.9]		0.50–1.86	1.32[1.0–1.8]		0.93[0.7–1.3]		0.61–2.00
Middle	0.99[0.8–1.2]		1.08[0.9–1.3]		0.88–1.28	0.94[0.8–1.1]		0.98[0.8–1.2]		0.86–1.09
High	Ref.		Ref.		Ref.	Ref.		Ref.		Ref.
**Asthma/COPD**		0.131		0.072			0.053		0.084	
Yes	0.93[0.7–1.3]		0.84[0.7–1.1]		0.61–1.59	0.86[0.5–1.4]		0.82[0.5–1.3]		0.63–1.03
No	Ref.		Ref.		Ref.	Ref.		Ref.		Ref.
**Sample**		0.735		0.816			<0.001		**<0.001**	
Low vaccination coverage sample	**0.68[0.5–0.9]**		**0.71[0.6–0.9]**		0.54–0.85	**0.73[0.6–0.9]**		**0.67[0.5–0.8]**		0.45–0.82
National sample	Ref.		Ref.		Ref.	**Ref.**		Ref.		Ref.
**Urbanization rate**		0.001		**0.004**			0.235		0.112	
Very high	**1.80[1.3–2.5]**		1.24[0.9–1.7]		0.57–1.96	1.08[0.8–1.4]		0.77[0.6–1.0]		0.66–0.93
High	1.40[0.9–2.2]		0.77[0.5–1.2]		0.45–1.80	1.11[0.9–1.4]		0.94[0.7–1.2]		0.79–1.22
Moderate high	0.97[0.8–1.3]		**0.73[0.6–0.9]**		0.62–1.00	0.97[0.7–1.3]		0.82[0.6–1.1]		0.62–1.15
Low	0.97[0.8–1.2]		0.85[0.7–1.0]		0.58–1.15	0.83[0.7–1.1]		1.03[0.9–1.3]		0.79–1.23
Very low	Ref.		Ref.		Ref.	Ref.		Ref.		Ref.

Bold indicates a p-value of <0.05. NA: Not available. Ref.: Reference.

**Table 2:** Geometric mean antibody concentration (GMC) ratios of elderly aged ≥65 years PIENTER1 and PIENTER2 based on univariable and multivariable generalized estimating equation analysis.

	PIENTER1					PIENTER2				
Determinants	Crude GMC ratio [95%CI]	P-value interaction- term crude analysis	Adjusted GMC ratio [95% CI]	P-value interaction-term adjusted analysis	Range of GMC ratio’s	Crude GMC ratio [95%CI]	P-value interaction- term crude analysis	Adjusted GMC ratio [95% CI]	P-value interaction-term adjusted analysis	Range of GMC ratio’s
**Contact with 0–4 year olds**	NA	NA	NA	NA	NA		0.375		0.343	
Contact						1.13[1.0–1.3]		1.05[0.9–1.3]		0.89–1.32
No contact						Ref.		Ref.		Ref.
**Contact with 5–19 year olds**	NA	NA	NA	NA	NA		0.465		0.507	
Contact						**1.16[1.0–1.3]**		1.14[1.0–1.3]		0.98–1.30
No contact						Ref.		Ref.		Ref.
**Contact with 20–59 year olds**	NA	NA	NA	NA	NA		0.429		0.125	
Contact						0.99[0.9–1.1]		0.96[0.9–1.1]		0.87–1.09
No contact						Ref.		Ref.		Ref.
**Contact with 60+ year olds**	NA	NA	NA	NA	NA		0.567		0.527	
Contact						0.97[0.9–1.1]		0.99[0.9–1.1]		0.88–1.08
No contact						Ref.		Ref.		Ref.
**Occupation with Children**	NA	NA	NA	NA	NA		0.138		0.275	
Yes						1.05[0.8–1.3]		1.05[0.8–1.3]		0.88–1.35
No						Ref.		Ref.		Ref.
**Household size**		0.005		**0.010**			0.941		0.924	
>2 persons	1.06[0.7–1.7]		1.01[0.7–1.6]		0.73–1.85	1.16[1.0–1.4]		1.09[0.9–1.3]		0.97–1.30
1–2 persons	Ref.		Ref.		Ref.	Ref.		Ref.		Ref.
**Gender**		0.558		0.814			0.774		0.853	
Male	1.06[0.9–1.3]		1.08[0.9–1.3]		0.83–1.33	**1.23[1.1–1.4]**		**1.26[1.1–1.4]**		1.09–1.35
Female	Ref.		Ref.		Ref.	Ref.		Ref.		Ref.
**Age**	1.01[1.0–1.0]	0.402	1.01[1.0–1.0]	0.401	0.98–1.03	**0.99[1.0–1.0]**	0.313	**0.99[1.0–1.0]**	0.326	0.97–1.00
**Ethnicity**		<0.001		**<0.001**			0.004		**0.035**	
Non-Western	**0.22[0.1–0.5]**		**0.33[0.2–0.5]**		0.08–2.00	1.15[1.0–1.4]		1.07[0.9–1.3]		0.81–1.54
Western	Ref.		Ref.		Ref.	Ref.		Ref.		Ref.
**Level of education**		0.012		0.129			0.436*		0.336	
Low	**1.63[1.2–2.2]**		**1.57[1.2–2.1]**		1.11–3.12	**1.21[1.1–1.4]**		**1.30[1.1–1.5]**		1.13–1.57
Middle	**1.42[1.0–1.9]**		**1.34[1.0–1.8]**		1.03–2.62	1.11[1.0–1.3]		1.14[1.0–1.3]		1.04–1.28
High	Ref.		Ref.		Ref.	Ref.		Ref.		Ref.
**Asthma/COPD**		0.740		0.636			0.015		**0.013**	
Yes	**1.54[1.1–2.1]**		**1.53[1.2–2.0]**		1.18–1.93	0.96[0.8–1.2]		0.95[0.8–1.2]		0.64–1.19
No	Ref.		Ref.		Ref.	Ref.		Ref.		Ref
**Sample**		0.644		0.700			0.231		0.208	
Low vaccination coverage sample	1.09[0.9–1.3]		**0.72[0.6–1.0]**		0.59–0.89	1.00[0.9–1.1]		1.00[0.8–1.2]		0.91–1.17
National sample	Ref.		Ref.		Ref.	Ref.		Ref.		Ref.
**Urbanization rate**		0.008		**0.029**			0.678		0.763	
Very high	**0.68[0.5–1.0]**		**0.57[0.4–0.9]**		0.32–0.96	0.96[0.8–1.1]		0.99[0.8–1.2]		0.87–1.14
High	**0.48[0.3–0.7]**		**0.43[0.3–0.7]**		0.24–0.64	1.10[1.0–1.3]		1.14[1.0–1.4]		1.05–1.24
Moderate high	**0.65[0.5–0.9]**		**0.54[0.4–0.8]**		0.42–0.73	0.98[0.8–1.2]		1.03[0.8–1.3]		0.85–1.20
Low	**0.72[0.6–0.9]**		**0.63[0.5–0.8]**		0.52–0.82	1.03[0.9–1.2]		1.07[0.9–1.3]		0.99–1.18
Very low	Ref.		Ref.		Ref.	Ref.		Ref.		Ref.

Bold indicates a p-value of <0.05. NA: not applicable. Ref.: Reference

* This estimate was calculated using an unstructured working correlation structure.

The analyses were carried out with SPSS version 19 (IBM SPSS Statistics 19, Chicago, USA).

## Results

### GMCs

**Fig 3:**
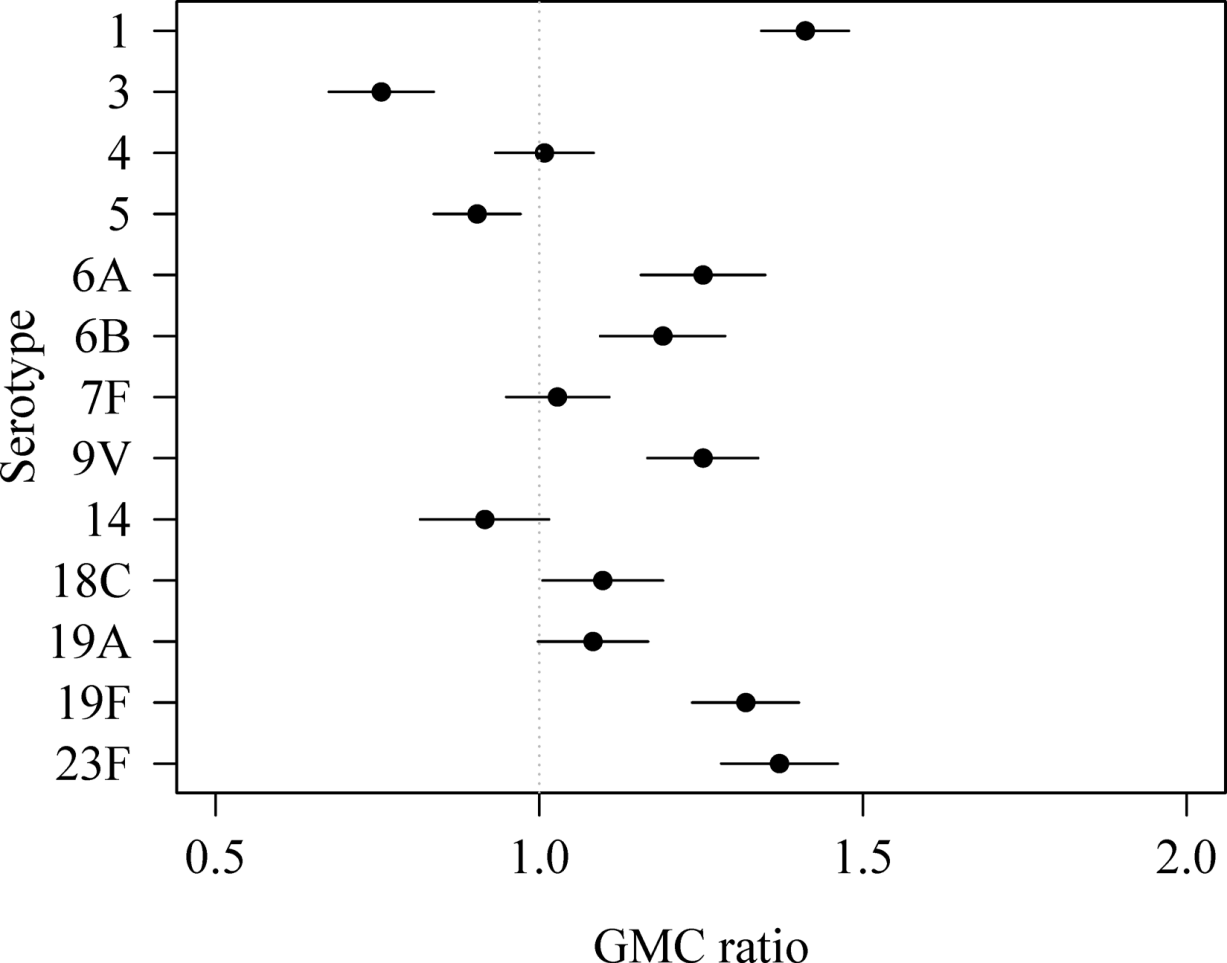
Weighted geometric mean concentration (GMC) ratios per serotype of PIENTER2 versus PIENTER1 with corresponding 95% confidence intervals.

For all age groups combined, the weighted GMCs of serotypes 1, 6A, 6B, 9V, 18C, 19F and 23F were significantly higher in PIENTER2 compared to PIENTER1 with weighted GMC ratios ranging between 1.19 for serotype 6B and 1.41 for serotype 1 (Fig 3). The weighted GMC ratios of serotypes 3 and 5 were significantly lower in PIENTER2 compared to PIENTER1 (GMC ratio 0.76 and 0.90, respectively).

**Fig 4:**
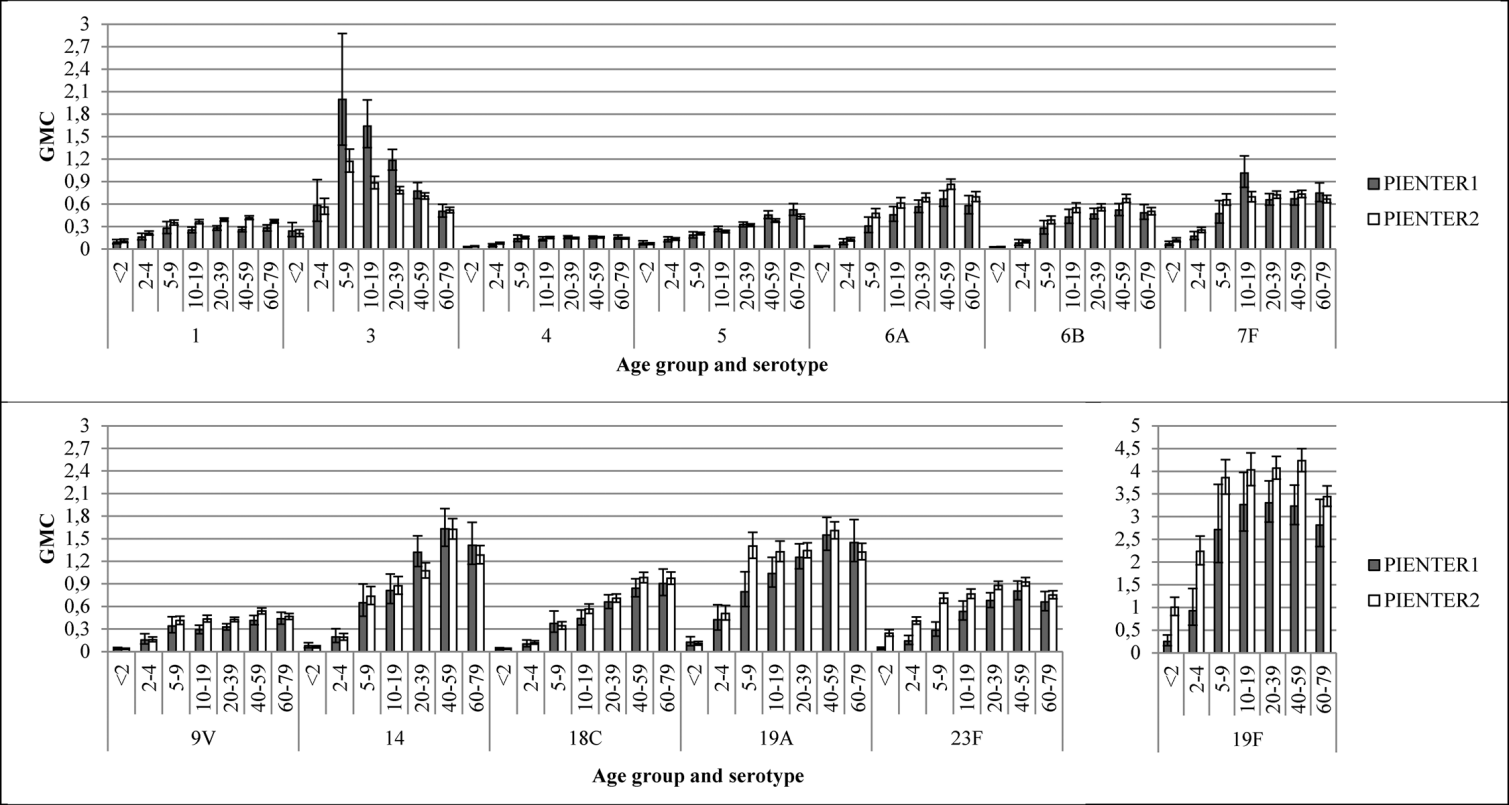
Weighted geometric mean concentrations (GMCs) with 95% confidence intervals of the national sample of PIENTER1 and PIENTER2 per serotype per age group.

The weighted GMC patterns per serotype for different age groups were comparable in PIENTER1 and PIENTER2 (Fig 4). In both studies an increase was seen from age group <2 years, weighted GMCs ranging from 0.02 for serotype 6B to 1.01 for serotype 19F, to age group 5–9 years, weighted GMCs ranging from 0.14 for serotype 4 to 3.86 for serotype 19F. For serotypes 1, 4,6A, 6B, 7F, 9V, 14, 19A, 19F and 23F the weighted GMCs were similar from this age group onwards (GMCs ranging from 0.13 for serotype 4 to 4.24 for serotype 19F), although for some of these serotypes (6A, 14, 19F and 23F) a small decrease in weighted GMC was seen after age group 40–59. The weighted GMCs of serotypes 5 and 18C continued to increase till the 60–79 age group, with weighted GMCs ranging from 0.44 for serotype 5 to 0.97 for serotype 18C. The weighted GMCs of serotype 3 increased till age group 5–9 after which the weighted GMCs declined in the following age groups.

**Fig 5:**
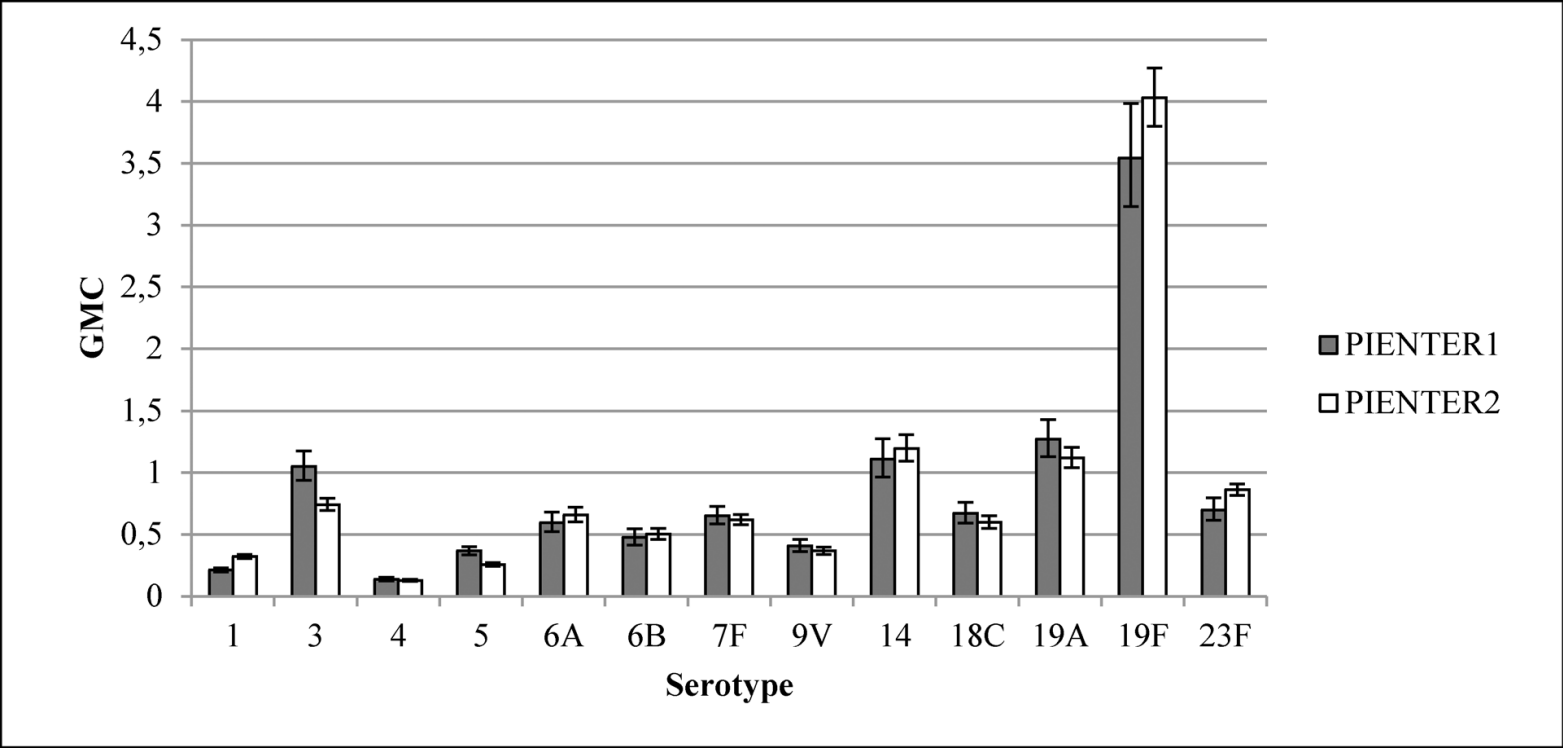
Weighted geometric mean concentrations (GMCs) with 95% confidence intervals of the low vaccination coverage sample of PIENTER1 and PIENTER2 per serotype.

Most weighted GMCs of the LVC samples were similar for PIENTER1 and PIENTER2. The weighted GMCs were significantly higher for serotypes 1, 19F and 23F and lower for serotypes 3 and 5 (Fig 5) in PIENTER 2 compared to PIENTER1.

## Associations between determinants and pneumococcal IgG concentration

In PIENTER2 a higher percentage of children attended day care (S1 Table). Also a higher percentage of participants had a non-western ethnicity and lived in areas with a high or very high urbanisation rate in PIENTER2.

The overall adjusted estimates for children in PIENTER1 showed that day care attendance of 0.5–2 days (GMC ratio 1.36, 95% CI 1.1–1.7) and increasing age (GMC ratio 1.42, 95% CI 1.3–1.6) were associated with a higher pneumococcal antibody concentration (Table 1). The sampling variable was significantly associated with pneumococcal IgG (GMC ratio 0.71 (95% CI 0.6–0.9)), indicating that there is a difference in pneumococcal IgG between participants living in areas with a low DTP-IPV vaccination coverage and participants living in areas with a high DTP-IPV vaccination coverage. A moderately high urbanization rate was significantly associated with a lower pneumococcal antibody concentration (GMC ratio 0.73, 95% CI 0.6–0.9). In the adjusted analysis of PIENTER2 a household size of 1–4 persons (GMC ratio 0.76, 95% CI 0.7–0.9) and the sampling variable (GMC ratio 0.67, 95% CI 0.5–0.8) were associated with a lower pneumococcal IgG concentration. Increasing age was significantly associated with a higher pneumococcal antibody concentration (GMC ratio 1.52, 95% CI of 1.4–1.6. The interaction terms of four determinants, that is frequency of day care attendance, age, ethnicity and urbanization rate, had p-values below 0.05 in the adjusted analysis of PIENTER1, meaning that the effects of these determinants differed between serotypes. Three determinants, household size, age and the sampling variable had a significant p-value for interaction in the adjusted analysis of PIENTER2. The ranges of the serotype-specific GMC ratios were not much larger for these determinants than for other determinants; also none of the serotypes had consistently the highest or lowest GMC ratio for the determinants with significant interaction terms.

For the elderly in PIENTER1, the overall estimates showed that having a non-western ethnicity, being in the LVC sample and living in an area with an urbanization rate other than very low were significantly associated with a lower pneumococcal antibody concentration in the adjusted analysis (Table 2). A low or middle education level and having asthma or COPD were associated with a higher pneumococcal antibody concentration. The adjusted analysis of participants aged ≥65 years of PIENTER2 showed that participants who were male (GMC ratio 1.26, 95% CI 1.1–1.4) and participants who had a low level of education (GMC ratio 1.30, 95% CI01.1–1.5) had a higher antibody concentration. Having a higher age (GMC ratio 0.99, 95% CI 1.0–1.0) was associated with lower pneumococcal antibody concentrations. The interaction terms of three determinants, household size, ethnicity and urbanization rate, had significant p-values in the adjusted analysis of PIENTER1. Two interaction terms, asthma/COPD and ethnicity, had significant p-values in the adjusted analysis of PIENTER2. Again, the ranges of the serotype-specific GMC ratios were not much larger for these determinants than for other determinants; also none of the serotypes had consistently the highest or lowest GMC ratio for the determinants with significant interaction terms.

## Discussion

This study compared the pneumococcal antibody levels and the determinants of these levels of two serosurveillance studies performed in the prevaccination period (1995–1996 and 2006–2007). We show that the GMCs were significantly different between the two studies for most of the pneumococcal serotypes targeted by PCV13. GMCs were higher in 2006–2007 for serotypes 1, 6A, 6B, 9V, 18C, 19F and 23F and lower for serotypes 3 and 5. Determinants of pneumococcal IgG were day care attendance, age, sampling variable, household size and urbanisation rate for children aged less than 4 years and urbanisation rate, level of education, gender, ethnicity, age, sampling variable and asthma/COPD for elderly. The determinants significantly associated with pneumococcal IgG were slightly different for the elderly in PIENTER1 compared to the elderly in PIENTER2. The directions of the associations were mostly the same in both PIENTER studies.

In the current study, an increase in GMC was seen until the age group 5–9 years. For the subsequent age groups the patterns of the GMCs could be categorised into three groups: 1) GMCs stayed approximately the same, 2) GMCs continued to increase till the 60–79 age group, 3) GMCs decreased after age group 5–9 years. A serosurveillance study was conducted in the United Kingdom (UK) in 2000–2004 in participants aged 0–93 years; this study described three patterns which are different from the patterns described in our study [15]. For instance, in the UK study most serotypes showed no increase in GMC between age 1 and 19, while in the current study the GMCs of all serotypes increased until age group 5–9 years. An explanation for the difference in patterns found in this study and the UK study might be the different sampling periods, but this seems unlikely since this study found no difference in patterns between PIENTER1 and PIENTER2. Therefore, the most likely explanation for the difference seems to be a different study population, either due to the sampling of study participants or regional (country) differences [15].

The presence of antibodies in an individual indicates that the individual has encountered the serotype at least once, but whether the serotype was carried or caused disease is not known [4]. Also, other determinants such as invasiveness and the ability to stimulate the human immune system can differ per serotype and influence the presence and level of pneumococcal antibodies. Taking this into account, we compared our results with those from carriage studies. A study of Bogaert et al. investigated pneumococcal carriage in Dutch children aged 3 months-3 years in 1999 (pre-PCV7). The serotypes carried mostly by these children were 6A, 6B, 9V, 14, 19F and 23F [16]. The serotypes with the highest GMCs in children in the current study and which are also described by Bogaert et al. are 14 (PIENTER1), 19F (PIENTER1 and PIENTER2), and 23F (PIENTER2). The difference could be explained by the different measurements (carriage vs. IgG) and the different time-period; this indicates that it is difficult to compare carriage and antibody levels; carriage of pneumococcal serotypes does not automatically result in an antibody response [16].

Most of the determinants of pneumococcal antibody concentrations found in this study are known risk factors for pneumococcal carriage or IgG antibody concentrations. For elderly, ageing and female gender are associated with a lower pneumococcal antibody concentration; whether these associations were present differed per serotype [17, 18]. Having asthma or COPD was related to a higher GMC in the PIENTER1 study, while having asthma was related to lower serotype specific antibodies in a previous study of Jung et al. Having asthma or COPD was not associated with pneumococcal antibodies in the PIENTER2 study, which might indicate that there are other factors, for instance time-period, influencing the association between asthma and pneumococcal antibodies [19]. A low/middle education level and a western ethnicity (only in the PIENTER1 study) were also associated with a higher pneumococcal antibody concentration in elderly in this study. These determinants were not yet reported for elderly, but have been associated with carriage or antibody concentrations in children [11, 20]. Contacts with children or working with children were not associated with pneumococcal antibody concentrations in elderly in our study. This was an unexpected finding since children are considered to be the reservoir of pneumococcus and therefore transmit the pneumococcus to the elderly [4, 12]. One explanation for this unexpected finding might be that the question about contacts with young children was not specific enough. Elderly might have had more often contact with older children, were carriage rate of *Streptococcus pneumoniae* is lower compared to young children.

The sampling variable was significantly associated with pneumococcal antibody levels in children of PIENTER1 and PIENTER2 and the elderly of PIENTER1. This indicates that vaccination coverage is associated with pneumococcal IgG even though the vaccination was added to the NIP in April 2006 and none of the participants included in the GMC calculations were vaccinated. Urbanisation rate was also associated with pneumococcal antibody levels for children and elderly in PIENTER1. The associations between vaccination coverage, urbanisation rate and pneumococcal IgG could be explained by community differences, for example household size and (type of) day care. Therefore, we expected that these associations would disappear when adding factors such as household size, ethnicity and frequency of day care attendance to the model, which was not the case, meaning that these factors did not (completely) explain the observed difference.

Attending a day care centre, living in a household consisting of more than four members and increasing age were statistically significantly associated with a higher antibody concentration in young children in our study; these determinants are known determinants for carriage of *Streptococcus pneumoniae* in children [21–23]. The determinants statistically significantly associated with pneumococcal IgG in children in PIENTER2 were similar as the determinants found in PIENTER1, which indicated that the determinants of pneumococcal encounters did not change over time in the pre-vaccine era. For the elderly the determinants associated with pneumococcal IgG in PIENTER1 were slightly different from the determinants found in PIENTER2, especially for ethnicity, asthma/COPD and urbanization rate, although due to the low number of elderly participants with a non-western ethnicity the association between ethnicity and pneumococcal IgG is less reliable. Studies which compared pre- and post-vaccine era determinants of pneumococcal carriage or IPD have found that some determinants remained a determinant irrespective of vaccination (ex. age) while other determinants disappeared [23–26].

One limitation of the PIENTER studies is that they had a cross-sectional design, therefore it is unknown whether the antibody concentrations really increased with increasing age or whether it is a cohort effect. Another limitation is the difference in response rate of the two studies, namely 55% for PIENTER1 and 34% for PIENTER2, this difference could result in selection bias, but using weight factors in the analysis of the GMCs and confounders in the GEE analysis we expect to have taken this into account as much as possible [7, 8]. A strength of the study is the large samples taken in the pre-vaccine era, making it possible to determine the dynamics of the pneumococcal serotypes before vaccination implementation.

In conclusion, during the pre-vaccine era the distribution of some serotypes changed while the determinants of pneumococcal antibodies remained similar. This indicates that the distribution of serotypes can change in the absence of widespread pneumococcal vaccination, which needs to be taken into account when determining the influence of vaccination on the dynamics of serotypes. Future studies should investigate pneumococcal antibody concentrations in the post-vaccine era to determine whether the distribution of serotypes and the determinants have changed after PCV implementation. When changes are found the results of the current study could contribute to determining what part was due to vaccination implementation and what part could be caused by natural fluctuations.

## Supporting Information

S1 Data GMC data underlying the figures in the article. (XLSX)

S2 Data Risk factor data of the children (0–3 years) PIENTER 1. (XLSX)

S3 Data Risk factor data of the elderly (≥65 years) PIENTER1. (XLSX)

S4 Data Risk factor data of the children (0–3 years) PIENTER 2. (XLSX)

S5 Data Risk factor data of the elderly (≥65 years) PIENTER2. (XLSX)

S1 Table Characteristics of children and elderly in PIENTER1 and PIENTER2. NA: Not available.
(DOCX)
