# Supplementary material for: Dynamics and Determinants of Pneumococcal Antibodies Specific against 13 Vaccine Serotypes in the Pre-Vaccination Era
Source: PLoS One. 2016 Jan 21;11(1):e0147437. doi: 10.1371/journal.pone.0147437 (PMC4721864; doi:10.1371/journal.pone.0147437)
Supplement: S1 Table — NA: Not available. (DOCX) [file pone.0147437.s006.docx]

| **Supplementary table.** Characteristics of children and elderly in PIENTER1 and PIENTER2. | | | | |
| --- | --- | --- | --- | --- |
| **Determinants** | **PIENTER1** | | **PIENTER2** | |
|  | 0-3 years  N (%) | ≥65 years  N (%) | 0-3 years  N (%) | ≥65 years  N (%) |
|  | N=372 | N=345 | N=642 | N=1174 |
| **Contact with 0-4 year olds**  >5 contacts  1-4 contacts  No contact | NA | NA | 118 (18.4)  215 (33.5)  309 (48.1) | 3 (0.3)  120 (10.2)  1051 (89.5) |
| **Contact with 5-19 year olds**  >5 contacts  1-4 contacts  No contact | NA | NA | 44 (6.9)  209 (32.6)  389 (60.6) | 26 (2.2)  242 (20.6)  906 (77.2) |
| **Contact with 20-59 year olds**  >5 contacts  1-4 contacts  No contact | NA | NA | 128 (19.9)  324 (50.5)  190 (29.6) | 235 (20.0)  458 (39.0)  481 (41.0) |
| **Contact with 60 +year olds**  >5 contacts  1-4 contacts  No contact | NA | NA | 7 (1.1)  144 (22.4)  491 (76.5) | 210 (17.9)  514 (43.8)  450 (38.3) |
| **Household member** ≤ **4 years**   ≥1 household member ≤ 4 years  No household member ≤ 4years | NA | NA | 256 (39.9)  386 (60.1) | NA |
| **Frequency of Day Care attendance**  >2 days  0.5-2 days  No attendance | 19 (5.1)  132 (35.5)  221 (59.4) | NA | 91 (14.2)  305 (47.5)  246 (38.3) | NA |
| **Occupation with Children**  Yes  No | NA | NA | NA | 79 (6.7)  1095 (93.3) |
| **Household size (children)**  1-4 persons  >4 persons | 275 (73.9)  97 (26.1) | NA | 435 (67.8)  207 (32.2) | NA |
| **Household size (elderly)**  >2 persons  1-2 persons | NA | 22 (6.4)  323 (93.6) | NA | 92 (7.8)  1082 (92.2) |
| **Gender**  Male  Female | 190 (51.1)  182 (48.9) | 157 (45.5)  188 (54.5) | 346 (53.9)  296 (46.1) | 598 (50.9)  576 (49.1) |
| **Ethnicity**  Non-Western  Western | 24 (6.5)  348 (93.5) | 2 (0.6)  343 (99.4) | 118 (18.4)  524 (81.6) | 89 (7.6)  1085 (92.4) |
| **Level of education**  Low  Middle  High | 8 (2.2)  185 (49.7)  179 (48.1) | 175 (50.7)  127 (36.8)  43 (12.5) | 38 (5.9)  347 (54.0)  257 (40.0) | 341 (29.0)  588 (50.1)  245 (20.9) |
| **Asthma/COPD**  Yes  No | 27 (7.3)  345 (92.7) | 39 (11.3)  306 (88.7) | 24 (3.7)  618 (96.3) | 94 (8.0)  1080 (92.0) |
| **Sample**  Low vaccination coverage sample  National sample | 102 (27.4)  270 (72.6) | 104 (30.1)  241 (69.9) | 173 (26.9)  469 (73.1) | 231 (19.7)  943 (80.3) |
| **Urbanization rate**  Very high  High  Moderate high  Low  Very low | 29 (7.8)  13 (3.5)  92 (24.7)  104 (28.0)  134 (36.0) | 29 (8.4)  22 (6.4)  79 (22.9)  103 (29.9)  112 (32.5) | 112 (17.4)  192 (29.9)  58 (9.0)  105 (16.4)  175 (27.3) | 189 (16.1)  434 (37.0)  129 (11.0)  127 (10.8)  295 (25.1) |
| NA: Not available. | | | | |
